# Medical and Philosophical Intelligence

**Published:** 1814-05

**Authors:** 


### MEDICAL AND PHILOSOPHICAL INTELLIGENCE.

ROYAL SOCIETY.—On Thursday the 17th of March, a paper by Dr. Crichton, of St. Petersburgh, was read, on the means by which vitality is supplied to the living system.

Dr. Crichton conceives that there is a continual waste of vitality during life, and therefore that a regular supply is necessary. He thinks that this vitality is furnished by the food, and believes that the food contains particles endowed with vitality, and that this vitality is neither destroyed by the destruction of the organic texture, nor by the heat to which the food is exposed. He made decoctions of camomile, feverfew, nutgalls, &c. in distilled water, put the decoctions into glass jars inverted over distilled mercury, and introduced into them oxygen gas obtained from black oxide of manganese. Numerous confervas made their appearance in these decoctions, and considerable portions of the gas were absorbed. From these experiments he concludes that there are two kinds of particles of matter, namely, organic particles and inorganic particles; and that the vitality of the first is not destroyed by boiling water. In general he found that vegetation commenced soonest when the decoction of flowers is used, and latest when that of roots.

*Case*

*Case of Anasarca* occurring in a man eighty-four years of age, cured, after many other methods had failed, by *frictions with powder of digitalis, mixed with saliva.*—Twenty grains of the powder to a coffee spoonful of saliva were rubbed in daily at first, and the quantity increased to forty grains. In about a week, a very copious flow of urine took place, and the patient sweated more than usual, He continued under this treatment about a month, and at length regained perfect health, in which state he has continued ever since, a period of nearly four years.—*Journal de Medecine.*

---

Mr. FORMAN, an ingenious glass-manufacturer upon the Wear, near Sunderland, has devised a convenient means for taking and preserving vaccine matter. The instrument is a small glass ball with a tube issuing from it, very similar to a cracker, as it is called, which mischievous boys put into candles to cause an explosion. The pustule from which the virus is to be taken being punctured by a lancet in the usual manner, the small ball or bulb is to be heated at a candle so as to rarefy the air within it, and after it is sufficiently warmed, the end of the little tube is to be inserted where the lancet had made the puncture, and the virus will immediately be taken up, so as to fill the bulb. The end of the tube is now to be hermetically sealed by means of a common blow-pipe at the flame of the candle, which is a very simple process; and thus the virus may be preserved for any length of time, and sent to any distance. If for immediate use, the tube need not be sealed, but may be secured in any convenient manner. Any requisite number of these balls may be employed, and it is proper to remark, that the virus is never heated much above blood heat.

---

*Fossil Human Skeleton,* placed among the minerals in the British Museum.—The mass of stone bearing the skeleton, is fixed nearly erect in one of the glass compartments: it is little more than four feet long, about two broad, and from four to nine inches thick. Although the head, neck, and feet are wanting, it is evident that the being to whom these bones belonged must have been of a stature rather less than men in general. The finger-bones of the left hand are situated so closely between those of the pelvis and thigh, being almost touching, that all the integuments must have been destroyed before these bones were enveloped with the calcareous matter. The block itself is a fine granular limestone, neither so compact as to appear uniformly crystallized, nor so porous as our common calcareous sandstones. There is some very similar limestone in the neighbourhood of Maidstone and West Malling, in Kent; the chief difference being that parts of the fragments of shells in this Guadaloupe stone are a bright red, while those in the Kentish stone are grey or yellow. Its fracture presents an appearance between that of a calcareous stone formed by simple deposition, and by imperfect crystallization; it is just such as might reasonably be expected to be formed in the vicinity of a volcano, where the solvent was considerably above the natural temperature of water, and much below that of metallic fusion.

---

M. J. J. VIREY has published in Paris a small pamphlet on the aphrodisiac aphrodisiac virtues of several plants, particularly of the one described in Holy Writ under the Hebrew name *Dudaim*, which Rachael, on a certain occasion, was so anxious to obtain. This word has been rendered *mandrake* in our version, but M. V. contends that the mandragora differs essentially from the plant called Dudaim ; and that in particular, this latter being described in the Songs of Solomon as bearing flowers of a very sweet smell, it cannot be the mandragora, whose flowers are completely of an opposite character. The author is said by the Parisian Reviewers to have demonstrated almost to evidence, from the etymology of the word, the period in which the plant flowers, its several botanic characters, and its physical and medical qualities, that the *Dudaim* is one of the *Orchis* family, and probably the same plant from which salep is prepared. *Vanilla*, obtained from a plant of this class, is known to possess similar powers, as is experienced by those who largely make use of chocolate.

---

On the 7th of April, Mr. Charles Bell, late Consulting-Surgeon to the Northern Dispensary, was elected one of the Surgeons to the Middlesex Hospital, in the place of the late Mr. Witham.

---

Mr. Joseph Hopkins will deliver a Course of Lectures on the Theory and Practice of Midwifery, at the Westminster Lying-in Institution, every month.

---

*Theatre of Anatomy, Bartlett's-court, Holborn.*—Mr. John Taunton, F.A.S. will commence his Summer Course of Lectures on Anatomy, Physiology, Pathology, and Surgery, on Saturday, May the 28th, 1814, at eight o'clock in the evening ; which will be continued every Tuesday, Thursday, and Saturday, at the same hour.

---

Mr. Carpue will commence his Lectures on Anatomy, Surgery, &c. early in June.

---

Dr. Davis will commence his Summer Course of Lectures on the Theory and Practice of Midwifery, and on the Diseases of Women and Children, on Monday the 6th of June, at nine o'clock.

